# Exploratory evaluation of spinopelvic and lumbar parameters in adolescent athletes with isthmic spondylolisthesis: feasibility and reliability of upright MRI compared to radiography

**DOI:** 10.3389/fradi.2026.1929174

**Published:** 2026-09-23

**Authors:** Michael Cassel, Theresa Joost, Chase Slater, Frank Mayer, Tilman Engel

**Affiliations:** University of Potsdam – Outpatient Clinic, Potsdam, Germany

**Keywords:** functional MRI, lower back, spinal instability, spondylolysis, x-ray

## Abstract

**Introduction:**

Isthmic spondylolisthesis is prevalent among adolescent athletes (AA). Upright MRI (uMRI) offers a radiation-free alternative to radiographs, combining imaging of the loaded spine with detailed depiction of the adjacent structures. This study evaluates the feasibility and intra-rater measurement repeatability of spinopelvic and lumbar parameter assessment in AA using uMRI and radiography, as well as cross-modality agreement between both imaging techniques.

**Material and methods:**

Lumbar sagittal T2-weighted low-field uMRI and lateral standing radiographs from 38 AA with isthmic spondylolisthesis were retrospectively reviewed. Cases of both imaging techniques without motion artifacts, performed within one year, presenting the lumbar spine and spinopelvic junction were included. Spinopelvic and lumbar parameters [e.g., anterior translation (AT)] were determined. Relative reliability was measured by intraclass correlation coefficient (ICC). Absolute reliability measures included standard error of measurement (SEM), test-retest variability (TRV), minimal detectable change (MDC), and Bland-Altman analysis.

**Results:**

Images from 16 AA (13.5 ± 1.8years; 159 ± 7 cm; 50 ± 9 kg) fulfilled the inclusion criteria. Data exclusion mainly occurred due to time discrepancy (*n* = 9) and poor image quality (*n* = 8). Relative reliability ranged from 0.89 to 0.95 for radiographs, 0.61 to 0.94 for uMRI and from 0.61 to 0.88 between the methods. Absolute reliability showed lower variability for radiographs (SEM: 3–8%; TRV: 5–14%, AT-MDC: 1.4 mm) compared to uMRI (SEM: 2–29%; TRV: 9–49%; AT-MDC: 2.0 mm) and between methods (SEM: 4–34%; TRV: 11–46%; AT-MDC: 3.2 mm).

**Discussion and conclusion:**

On evaluable images, uMRI demonstrated moderate-to-excellent intra-rater measurement repeatability for several parameters. However, cross-modality agreement with radiography was variable and does not support interchangeability between the two modalities. uMRI may therefore represent a complementary radiation-free imaging modality for selected patients and parameters, pending prospective validation.

## Introduction

Low back pain is increasingly prevalent in young populations (1), specifically among adolescent athletes (AA) cohorts. A significant contributing factor to this is observed with isthmic spondylolisthesis, which has an estimated prevalence of 4%–8% by the time individuals reach adulthood (1). Marchetti-Bartolozzi identified six primary aetiologies for spondylolisthesis: Developmental (dysplastic), isthmic, traumatic, degenerative, pathologic, and post-operational (1, 2). Among these aetiologies, isthmic spondylolisthesis demonstrates a strong association with high levels of physical activity and occurs as a progression of spondylolysis, characterized by a bilateral pars interarticularis defect (1, 3). This defect potentially leads to an anterior translation of the superior vertebral segment to the inferior vertebral segment, commonly occurring at the L5/S1 level (1, 4). The degree of slippage or forward translation of the vertebral segment in spondylolisthesis is typically assessed via standing radiography using the Meyerding Classification: grade I (0%–25%), grade II (25%–50%), grade III (50%–75%), grade IV (75%–100%), and grade V (>100%) (1–3). The occurrence of isthmic spondylolisthesis is often correlated to movement patterns emphasizing lumbar reclination and rotation, observed more frequently in sports, like javelin throwing or gymnastics, involving repetitive, extensive, and compressive forces acting on the spine and pars interarticularis (1, 3).

When an athlete presents with persistent low back pain, in clinical practice, a routine radiograph is usually taken as a first step in evaluation (1, 4). Upright, lateral radiographs performed in two planes are considered the current gold standard for diagnosing spondylolisthesis. Alternatively, upright magnetic resonance imaging (uMRI) could be able to detect the bony displacement and additionally provide improved visualization of other potential factors contributing to low back pain, including pars edema, disc and facet joint degeneration, stenosis, compromised musculo-ligamentous integrity and paravertebral muscle atrophy (5, 6). While MRI is generally utilized in patients with neurological symptoms (4), its application for isthmic spondylolisthesis in an upright, weight-bearing position has not been extensively studied (5). The available evidence comparing both imaging modalities for diagnosing and grading the pathology and quantifying potential segmental vertebral slippage is limited (7, 8). Previous studies regarding isthmic spondylolisthesis have clinically compared conventional MRI to uMRI as well as uMRI to standard radiographs (8, 9). However, reliability and feasibility investigations to determine a measurement error of the techniques were not performed concerning spinopelvic and lumbar parameters in young patients with isthmic spondylolisthesis. In particular, intra-rater measurement repeatability, reflecting the consistency of repeated measurements of the same images by the same examiner, remains insufficiently investigated. Similarly, cross-modality agreement between radiography and uMRI in a young athletic population remains unclear (10). This study therefore aims to evaluate the intra-rater measurement repeatability of spinopelvic and lumbar parameters using radiographs and uMRI in AA with isthmic spondylolisthesis, the agreement between modalities, and the feasibility of uMRI for clinical application.

## Material and methods

A cohort of 38 AA (13.4 ± 1.9 years, 162.4 ± 12.1 cm, 52.8 ± 13.9 kg) with isthmic lumbar spondylolisthesis diagnosed during a medical preparticipation examination (October 2009 to June 2019) at the University Outpatient Clinic determining eligibility for performing competitive sports (Table 1) were recruited for inclusion in this retrospective analysis. Radiography was performed during preparticipation examination. The subsequent uMRI imaging was acquired in an additional study in collaboration with a local hospital. The participants and the legal guardians of the minors provided written consent for participation in the study, which was approved by the local Ethics Committee of the University and complies with the guidelines for conducting clinical studies by the Declaration of Helsinki.

**Table 1:** Athletic background of participants (*n* = 38).

Type of sport	Number of participants	Proportional share in relation to total population [%]
Wrestling	9	23.7
Canoe	7	18.4
Track and Field	7	18.4
Judo	6	15.8
Swimming	3	7.9
Weightlifting	1	2.6
Rowing	1	2.6
Horseback Riding	1	2.6
Soccer	1	2.6
Road Cycling	1	2.6
Gymnastics	1	2.6

### Imaging data

Lateral standing radiographs and sagittal T2-weighted uMRI at 80° were obtained from the database. For uMRI acquisition, participants were positioned in a standardized weight-bearing position with the upper body and pelvis were stabilized using dedicated straps, the knees were supported by a knee roll, and the feet positioned hip-width apart in a neutral position. The arms were positioned at approximately 90° of flexion and supported on the MRI system to provide additional stabilization during image acquisition. All radiographic examinations were acquired in an unsupported upright standing position (90°) using the same radiography system (Philips Bucky Diagnost Version 1, University of Potsdam Outpatient Clinic; standardized focus-to-detector distance: 150 cm), performed by five different operators. Likewise, all uMRI examinations were acquired using the same device and scan protocol (0.25 T, Esaote G-Scan, Italy, Genova; Sequences: FSE, time to echo: 125 ms, slice thickness: 4 mm, flip angle: 90°, time to repetition: >2,200 ms), performed by two different operators (two radiological technicians, highly experienced in sequence acquisition of the uMRI system). To be included in the comparison, the radiographs and uMRI scans had to have been taken within one year and show the entire lumbar spine from L1 to the lumbopelvic junction without motion artifacts.

### Data processing

Image eligibility and quality were assessed by a single examiner (health scientist and physiotherapist with 10 years of clinical experience in orthopedics and five years of experience in medical image acquisition and evaluation) based on image availability, the time interval between acquisition of the two modalities, and sufficient visualization of the anatomical landmarks required for measurement. Images were considered of insufficient quality when relevant anatomical landmarks or characteristic reference points required for calculation of the respective parameters could not be clearly identified, for example due to motion artifacts. The spinopelvic and lumbar parameters were assessed twice (M1 and M2) on the same radiographic and uMRI images by the same examiner with an interval of at least seven days between measurements. Thus, M1 and M2 represent repeated measurements of the same images, and the within-modality analyses assess intra-rater measurement repeatability. No repeated image acquisition was performed. Re-evaluation of the images (M2) was performed in the identical order at least seven days after the initial assessment (M1), with the examiner blinded to the previous measurement results and clinical information.

The following parameters were assessed (11):

1. Anterior translation [AT, (mm)]: distance between tangent placed on posterior margin of L5 and superior endplate of S1
2. Lumbosacral joint angle [LSJA, (°)], angle between the cranial endplate of S1 and the caudal endplate of L5
3. Lumbar lordosis angle [LLA, (°)], angle between cranial endplate S1 and cranial endplate L1Sacral slope angle
4. Sacral slope angle [SS, (°)] angle between superior endplate of S1 and the horizontal
5. Intervertebral disc height [IVDH, (mm)]: mean value of the distance between the cranial cover plate S1 and the caudal cover plate of L5, measured at 3 intersegmental positions: anterior, middle, posterior

Local segmental (IVDH, SS), translational (AT) and rotational (LSJA) as well as global (LL) parameters were selected for comprehensive visualization of the spine and the lumbopelvic junction and its configuration (11).

### Statistical analysis

Data analysis included a descriptive analysis for all anthropometrics, spinopelvic, and lumbar parameters (mean, 95% Confidence Interval, CI). Intra-rater measurement repeatability was assessed using the intraclass correlation coefficient (ICC; Model 3,1; absolute agreement) for repeated measurements of the same images within each modality (radiograph: M1_R vs. M2_R; uMRI: M1_M vs. M2_M). ICC values were calculated using SPSS, version 28. ICC grading was based on Koo and Li (2016), where >0.9 represents excellent; 0.75–0.9 represents good; 0.5–0.75 represents moderate; and <0.5 represents poor results (12). Absolute measures of intra-rater measurement repeatability were calculated on Microsoft Excel (version 2306) represented by the standard error of measurement (SEM, [SD * square root of (1–ICC)]; SEM%, [(SEM/mean) *100]), the test-retest variability (TRV%, [(|x_i_–y_i_|/0.5 (x_i_+y_i_) *100), where x_i_ represents the values of measurement 1 and y_i_ those of measurement 2 for subject _i_]), minimal detectable change {MDC, [1.96 * square root of (2) * SEM]}, and through a Bland-Altman analysis, including bias, upper (uLoA = bias +1.96*SD), and lower limits of agreement (lLoA = bias −1.96*SD) (13). Cross-modality agreement between radiography and uMRI was assessed separately using ICC and Bland–Altman analysis in the paired cohort (*n* = 16).

## Results

### Feasibility and cohort characteristics

The available sample size differed between analyses according to the availability of evaluable measurements for the respective imaging modality. The intra-rater reliability analyses comprised 29 radiographic and 20 uMRI datasets, respectively. Of the original cohort of 38 adolescent athletes, 16 (42%) fulfilled the eligibility criteria for the paired cross-modality comparison of radiography and uMRI. The participant selection process and reasons for exclusion from the paired analysis, including insufficient image quality, incomplete uMRI sequences, an examination interval exceeding one year, and absence of identifiable anterior vertebral displacement consistent with spondylolisthesis, are summarized in Figure 1.

**Figure 1:**
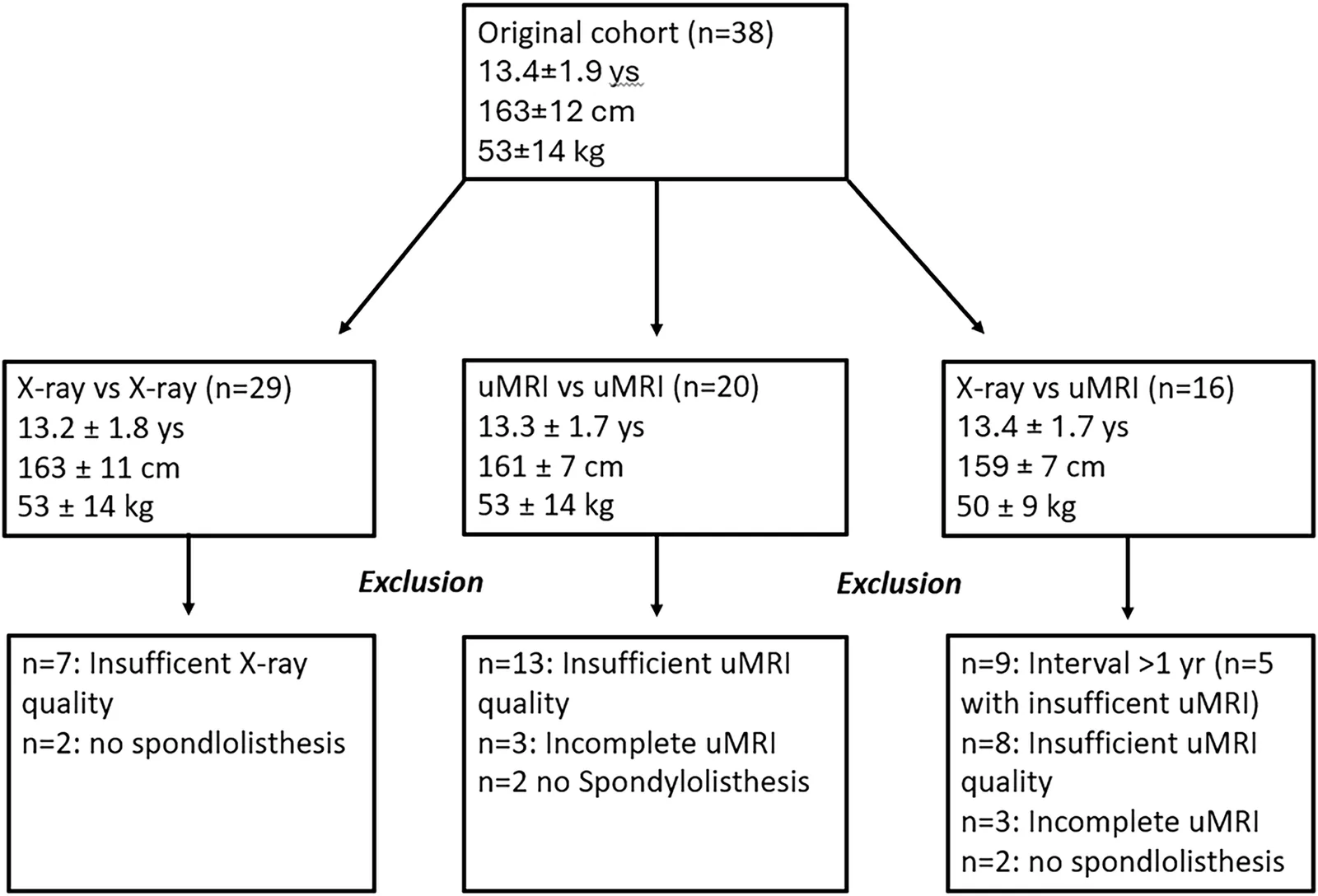
Study flow chart illustrating reasons for exclusion of different cohorts analyzed regarding x-ray (radiography) and uMRI images.

The final paired cohort comprised 10 female and 6 male athletes with a mean age of 13.4 ± 1.7 years (range 11–17 years), a mean height of 159.2 ± 6.7 cm, and a mean weight of 49.6 ± 9.0 kg. All participants showed spondylolisthesis at L5/S1 level, Meyerding grade 1 was detected in twelve and grade 2 in four cases. The pain level on a visual analog scale was reported to be 2.0 ± 2.8 during sports. The mean interval between radiography and uMRI was 5.2 ± 3.9 months. Descriptive statistics of the spinopelvic and lumbar parameters are presented in Table 2.

**Table 2:** Descriptive statistics for spinopelvic and lumbar parameters (mean [95% CI; *n* = 16).

Parameter	M_1_R_	M_2_R_	M_1_M_	M_2_M_
AT [mm]	8.6 [7.3–9.8]	8.6 [7.4–9.9]	6.7 [5.8–7.6]	6.9 [6.0–7.8]
LSJA [°]	10.5 [8.3–12.7]	10.3 [7.8–12.9]	9.1 [6.6–11.5]	8.6 [6.1–11.0]
LLA [°]	65.2 [59.7–70.6]	65.0 [60.2–69.9]	61.2 [54.3–68.1]	60.6 [54.9–66.3]
SS [°]	49.2 [45.5–53.0]	48.2 [44.3–52.1]	47.5 [42.6–52.3]	45.8 [41.6–50.1]
IVDH [mm]	9.2 [8.5–9.9]	8.5 [7.7–9.3]	6.9 [6.4–7.5]	7.1 [6.6–7.6]

AT, Anterior translation [mm]; LSJA, Lumbosacral joint angle [°]; LL, Lumbar lordosis [°]; SS, Sacral slope [°]; IVDH, Intervertebral disc height [mm]; M_1_, Measurement time point 1; M_2_, Measurement time point 2; R, Radiographs; M, uMri.

### Intra-rater measurement repeatability

The relative reliability ranged from ICC_IVDH_ = 0.89 to ICC_AT_ = 0.95 using radiograph scans (*n* = 29) and from ICC_IVDH_ = 0.61 to ICC_SSA_ = 0.94 using uMRI scans (*n* = 20). In terms of absolute reliability, TRV ranged from TRV_LLA_ = 5% to TRV_LSJA_ = 14%; SEM ranged from SEM_SS_ = 2% to SEM_LSJA_ = 8%; MDC ranged from MDC_IVDH_ = 1.4 mm to MDC_AT_ = 1.5 mm using radiographs and TRV ranged from TRV_SS_ = 9% to TRV_LSJA_ = 49%; SEM ranged from SEM_SS_ = 2% to SEM_LSJA_ = 29%; MDC ranged from MDC_AT_ = 2.0 mm to MDC_IVDH_ = 2.4 mm using uMRI. No indication for systematic error for both devices based on Bland-Altman analysis. Details are shown in Table 3 and Figure 2.

**Table 3:** Absolute and relative intra-rater measurement repeatability analysis of spinopelvic parameter.

Diagnostic method	Statistical measure	AT	LSJA	LLA	SS	IVDH
Radiograph vs. Radiograph (*n* = 29)	ICC (95% CI)	0.95 (0.90–0.98)	0.93 (0.86–0.97)	0.95 (0.89–0.98)	0.95 (0.90–0.98)	0.89 (0.67–0.96)
	TRV [%]	11	14	5	5	8
	SEM	0.3 mm	0.7°	1.0°	0.8°	0.3 mm
	SEM [%]	3	8	2	2	3
	Bias	−0.2 mm	0.2°	0.02°	0.9°	0.5 mm
	lLoA	−2.8 mm	−5.2°	−8.8°	−6.5°	−1.0 mm
	uLoA	2.4 mm	5.6°	8.9°	8.3°	2.0 mm
	MDC	1.5 mm	2.4°	2.8°	2.6°	1.4 mm
uMRI vs. uMRI (*n* = 20)	ICC (95% CI)	0.87 (0.70–0.95)	0.74 (0.36–0.90)	0.90 (0.77–0.96)	0.94 (0.86–0.98)	0.61 (0.09–0.84)
	TRV [%]	19	49	11	9	13
	SEM	0.5 mm	2.7°	2.7°	1.1°	0.8 mm
	SEM [%]	8	29	4	2	11
	Bias	−0.4 mm	0.9°	0.4°	1.7°	−0.4 mm
	lLoA	−3.2 mm	−9.4°	−16.2°	−7.4°	−2.8 mm
	uLoA	2.4 mm	11.1°	16.9°	10.9°	1.9 mm
	MDC	2.0 mm	4.6°	4.5°	3.0°	2.4 mm

AT, Anterior translation [mm]; LSJA, Lumbosacral joint angle [°]; LLA, Lumbar lordosis angle [°]; SS, Sacral slope [°]; IVDH, Intervertebral disc height [mm]; ICC, Intraclass correlation coefficient; TRV, Test-retest variability; SEM, Standard error of measurement; lLoA, Lower limit of agreement; uLoA, Upper limit of agreement; MDC, Minimal detectable change.

**Figure 2:**
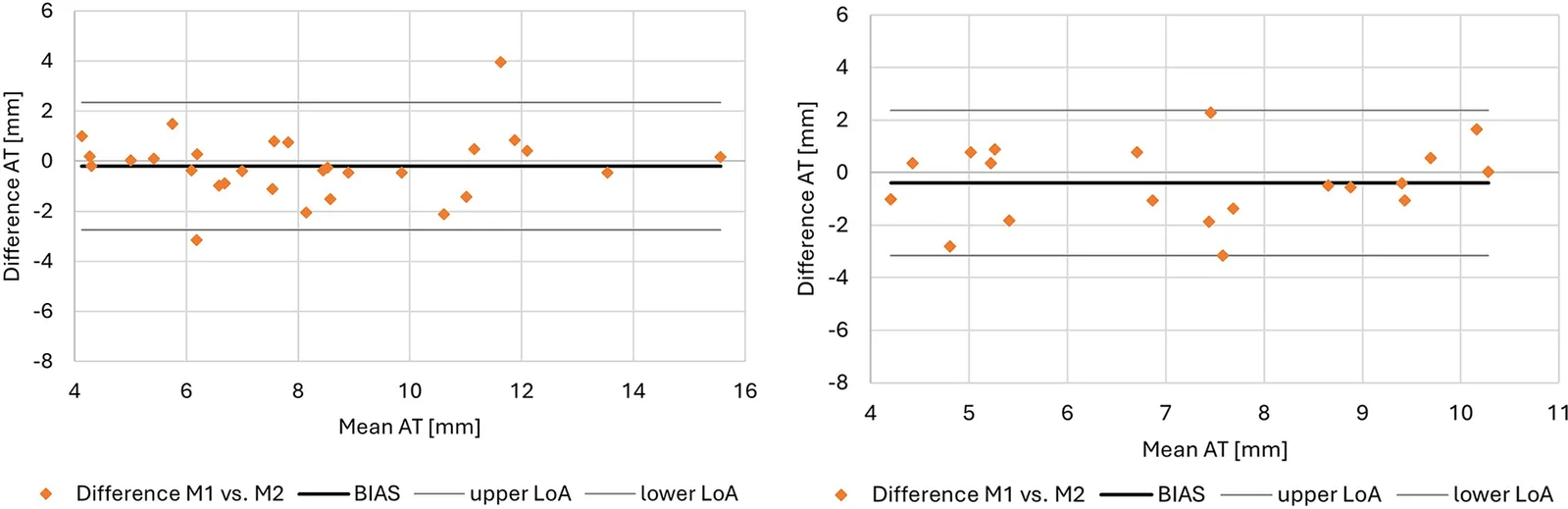
**(a)** Bland-altman plots, intra-rater reliability of AT in radiographs (*n* = 29 included radiographs). **(b)** Bland-Altman plots, intra-rater reliability of AT in uMRI (*n* = 20 included uMR images).

For radiographic measurements, the highest reliability was observed for AT (ICC = 0.95), while the lowest reliability was found for IVDH (ICC = 0.89). The TRV ranged from 5% (LLA) to 14% (LSJA), while SEM values ranged from 2% (SSA) to 8% (LSJA). The MDC values were between 1.4 mm (IVDH) and 1.5 mm (AT).

For uMRI, the highest ICC value was found for SSA (ICC = 0.94), while the lowest was for IVDH (ICC = 0.61). TRV ranged from 9% (SSA) to 49% (LSJA), with SEM values between 2% (SSA) and 29% (LSJA). The MDC values ranged from 2.0 mm (AT) to 2.4 mm (IVDH). No systematic error was detected for either imaging modality based on Bland-Altman analysis (Table 3).

### Agreement between radiographs & uMRI

The ICC for agreement between radiographs and uMRI measurements ranged from ICC_AT_ = 0.61 to ICC_LLA_ = 0.88. The absolute reliability, TRV ranged from TRV_LLA_ = 11% to TRV_LSJA_ = 46%; SEM ranged from SEM_LLA_ = 4% to SEM_LSJA_ = 34%; MDC ranged from MDC_IVDH_ = 2.8 mm to MDC_AT_ = 3.2 mm between radiographs and MRI. Details are shown in Table 4 and Figure 3.

**Table 4:** Cross-modality agreement between 2 examination methods of spinopelvic parameter.

Diagnostic method	Statistical measure	AT	LSJA	LLA	SS	IVDH
Radiograph vs. uMRI (*n* = 16)	ICC (95% CI)	0.61 (−0.8–0.86)	0.65 (0.04–0.87)	0.88 (0.60–0.96)	0.82 (0.50–0.94)	0.53 (−0.19–0.83)
	TRV [%]	26	46	11	15	21
	SEM	1.3 mm	3.3°	2.7°	3.2°	1.0 mm
	SEM [%]	18	34	4	7	14
	Bias	−1.6 mm	−1.8°	−4.3°	−2.6°	−1.4 mm
	lLoA	−5.7 mm	−12.7°	−19.3°	−17.5°	−4.3 mm
	uLoA	2.6 mm	9.1°	10.7°	12.4°	1.6 mm
	MDC	3.2 mm	5.0°	4.5°	5.0°	2.8 mm

AT, Anterior translation [mm]; LSJA, Lumbosacral joint angle [°]; LLA, Lumbar lordosis angle [°]; SS, Sacral slope [°]; IVDH, Intervertebral disc height [mm]; ICC, Intraclass correlation coefficient; TRV, Test-retest variability; SEM, Standard error of measurement; lLoA, Lower limit of agreement; uLoA, Upper limit of agreement; MDC, Minimal detectable change.

**Figure 3:**
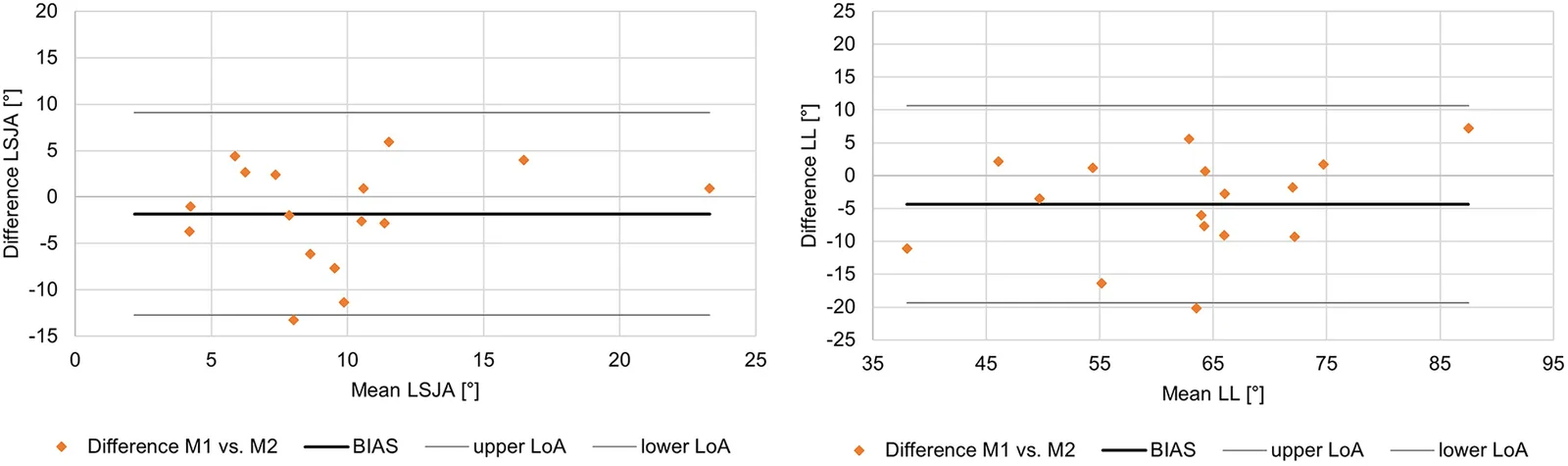
**(a)** Bland-altman plots, intra-rater reliability of LSJA between devices (*n* = 16). **(b)** Adjustments in Bland-Altman plots, intra-rater reliability of LLA between devices (*n* = 16).

The highest agreement was observed for LLA (ICC = 0.88), whereas AT showed the lowest agreement (ICC = 0.61). TRV values ranged from 11% (LLA) to 46% (LSJA). The SEM values varied from 4% (LLA) to 34% (LSJA), while MDC ranged from 2.8 mm (IVDH) to 3.2 mm (AT). Detailed agreement statistics are presented in Table 4, Figure 3.

## Discussion

This study evaluated the feasibility and intra-rater measurement repeatability of spinopelvic and lumbar parameter assessment using upright radiography and uMRI in adolescent athletes with isthmic spondylolisthesis, as well as cross-modality agreement between both imaging techniques. On selected, evaluable images, intra-rater measurement repeatability was good to excellent for most radiographic parameters and ranged from moderate to excellent for uMRI. However, agreement between radiography and uMRI was parameter-dependent and showed substantial variability, limiting conclusions regarding interchangeability between the two modalities.

Kanno et al. (8) analyzed the diagnosis rate of degenerative spondylolisthesis in an axial-loaded (upright) and supine MRI in patients with degenerative spondylolisthesis. The authors also reported a reduced diagnosis rate in the unloaded position (8). Alvi et al. (14) explored spinopelvic parameters by comparing radiography to supine MRI in 255 patients with degenerative spondylolisthesis and found poor agreement between the two modalities for AT (14). In line with these previous findings, our results demonstrate that measurements obtained from radiography and uMRI should not be assumed to be interchangeable, as agreement varied considerably across parameters.

### Feasibility

Thirteen (34%) out of 38 uMRI scans were illegible for the analysis due to inadequate image quality. This suggests that using the available uMRI at low-field rate with 0.25 Tesla limits the assessment of isthmic spondylolisthesis in this cohort. Specifically motion artefacts in young AA during standing position of more than 10 min led to inaccurate image quality and an increased data exclusion rate. In clinical practice, usage of MRI scanners with higher field strength (minimum of 1.5 Tesla) is strongly recommended to identify relevance of slippage and concomitant pathologies involving surrounding structures under loading more precisely and regularly (8, 15). Moreover, examination time of upright sequences should be reduced below 10 min among AA.

### Intra-rater measurement repeatability

The intra-rater analyses indicate that, on selected images of sufficient quality, a single examiner could reproduce several spinopelvic and lumbar measurements with reasonable consistency. Repeatability was generally higher for radiographic measurements (ICC = 0.89–0.95) than for uMRI (ICC = 0.61–0.94), which may reflect the established clinical familiarity with radiographic assessment and the potential challenges in measuring and interpreting uMRI scans. Absolute measures of repeatability also indicated greater measurement variability for uMRI, with TRV values of up to 49% and MDC values of up to 2.4 mm, compared with TRV values of 5%–14% and MDC values of 1.4–1.5 mm for radiographs. No indication of systematic measurement error was observed for either modality across the assessed local and global parameters. Importantly, these findings reflect intra-rater measurement repeatability based on repeated assessment of the same images and should not be interpreted as evidence of scan–rescan reliability or diagnostic performance of either imaging modality.

### Agreement between radiographs and uMRI

The exploratory cross-modality analysis showed parameter-dependent agreement between radiography and uMRI (ICC = 0.53–0.88). These modalities may produce relatively comparable measurements in some cases, but there can be notable discrepancies as well. This is evident in cases of local parameters (AT, LSJA and IVDH). The increased variability and lower agreement (e.g., LSJA) between radiographs and uMRI further underscore the potential for positional differences and imaging artifacts to affect diagnostics outcomes. Importantly, the relatively high ICC observed for some parameters should therefore not be interpreted independently of the corresponding bias and limits of agreement, as relative agreement does not necessarily indicate sufficient absolute agreement between modalities. Contrarily, LSJA has been shown to be the most reliable measurement in a study by Okpala (2019) to determine lumbar lordosis in healthy spines (16). Global parameters, particularly the lumbar lordosis angle, also exhibit systematic errors. A bias of −4.3° was observed in uMRI compared to radiographs, highlighting the importance of considering these deviations in clinical assessments.

### Limitations

Several methodological and technical limitations should be considered when interpreting the present findings. Upright MRI with the available scanner required participants to remain standing still for approximately 10 min during T2-weighted imaging. The prolonged acquisition time increased the susceptibility to motion artifacts. Although participants were positioned at an 80° inclination to reduce fatigue, minor movements may have compromised image clarity and measurement precision. In addition, the low field strength of 0.25T limited image resolution compared with higher-field MRI systems.

Despite the retrospective design, image acquisition was standardized in several respects: all examinations within each modality were acquired using the same respective imaging system and according to the established institutional imaging setup. Nevertheless, radiographic and uMRI examinations were performed by five and two different operators, respectively, and operator-related variability in image acquisition therefore cannot be excluded. Although radiographs were acquired using a standardized focus-to-detector distance of 150 cm, information on image-specific calibration or correction for radiographic magnification was not available retrospectively. Therefore, potential magnification effects on linear radiographic measurements, particularly AT and IVDH, cannot be excluded. Due to the retrospective nature of the study, some additional technical metadata requested for full methodological characterization, including field of view, matrix, in-plane resolution, coil specifications, and exact acquisition duration, were no longer available.

Although positioning was standardized within each imaging modality, it was not equivalent between modalities. uMRI was performed at an 80° inclination with stabilization of the upper body and pelvis, knee support, and arm support, whereas radiographs were acquired in an unsupported upright standing position at 90°. These differences in positioning and external support may have influenced spinal alignment and therefore contributed to the observed differences between modalities.

The relatively small sample size of the paired cross-modality analysis (*n* = 16) represents an important limitation, restricting both the precision and generalizability of the findings. Specifically, the ICC estimates representing relative reliability values should be generally interpreted with caution in small samples. Eight participants were excluded from the paired analysis due to insufficient uMRI image quality and nine because the interval between radiography and uMRI exceeded one year. Restricting the paired analysis to examinations with sufficient image quality may have introduced selection bias and may have resulted in an overestimation of measurement repeatability and cross-modality agreement compared with routine clinical imaging. Moreover, developmental changes during the interval between examinations may have affected spinal morphology. Consequently, the observed differences between radiography and uMRI cannot be attributed solely to the imaging modality.

### Clinical implications and future research

The findings of this study provide initial information on the potential complementary role of uMRI in the assessment of isthmic spondylolisthesis in adolescent athletes. On evaluable uMRI scans, AT showed good intra-rater measurement repeatability (ICC = 0.87); however, cross-modality agreement with radiography was only moderate (ICC = 0.61) and absolute agreement remained limited. Thus, the present findings do not support interchangeability between uMRI and radiography for AT assessment. Radiography should therefore remain the reference method for diagnosis and grading. Nevertheless, uMRI may provide complementary information by enabling assessment under weight-bearing conditions while simultaneously visualizing surrounding structures. Beyond the quantitative assessment of vertebral slippage investigated in the present study, uMRI may additionally identify findings such as bone marrow edema, disc pathology, or other soft-tissue abnormalities that may contribute to symptoms (17).

Rotational alignment, specifically assessed through LSJA, and some global parameters, such as the lumbar lordosis angle, presents more challenges. LSJA exhibited moderate agreement between modalities (ICC = 0.65), with radiographs providing greater measurement precision (TRV = 14%, MDC = 2.4°) compared to uMRI (TRV = 46%, MDC = 4.6°). Similarly, LLA measurements demonstrated a systematic error, with a bias of −4.3° in uMRI compared to radiographs, reinforcing the need for cautious interpretation. Given these discrepancies, radiographs should at present remain the preferred method for LSJA and LLA assessments, while uMRI may provide complementary information in selected patients and for selected parameters. Future prospective validation studies should employ same-day imaging, standardized and equivalent positioning across modalities, and preferably randomized modality order. Further standardization of uMRI acquisition and measurement protocols for spinopelvic and lumbar parameters, as well as studies in larger and more diverse populations, will be essential to provide more robust estimates of measurement repeatability and cross-modality agreement and to further evaluate the potential clinical role of uMRI.

## Conclusion

While radiographs remain the reference standard for assessing isthmic spondylolisthesis, uMRI shows promise as a complementary tool for the assessment of selected spinopelvic and lumbar parameters. On evaluable uMRI scans, a single examiner demonstrated moderate-to-excellent intra-rater measurement repeatability for several parameters. However, agreement with temporally separated radiographs was variable and does not support interchangeability between the two modalities. The feasibility of uMRI in clinical practice is influenced by factors such as image quality, acquisition time, and patient cooperation, particularly when using the low-field uMRI system investigated in this study. Although the small sample size and the retrospective study design limit the precision and generalizability of the findings, this study provides a basis for future prospective investigations using uMRI-systems with higher field strength.

## Data Availability

The original contributions presented in the study are included in the article/Supplementary Material, further inquiries can be directed to the corresponding author/s.
